# Work-Related Musculoskeletal Disorders among Nurses in Ibadan, South-west Nigeria: a cross-sectional survey

**DOI:** 10.1186/1471-2474-11-12

**Published:** 2010-01-20

**Authors:** Bolanle MS Tinubu, Chidozie E Mbada, Adewale L Oyeyemi, Ayodele A Fabunmi

**Affiliations:** 1 Physiotherapy department, University College Hospital, Ibadan, Nigeria; 2 Department of Medical Rehabilitation, College of Health Sciences, Obafemi Awolowo University, Ile - Ife. Nigeria; 3 Department of Physiotherapy, University of Maiduguri, College of Medical Sciences, Maiduguri, Nigeria; 4 Physiotherapy department, College of Medicine, University of Ibadan, Nigeria

## Abstract

**Background:**

Musculoskeletal disorders represent a significant occupational problem among nurses; however, data on musculoskeletal health of nurses in Sub-Sahara Africa are sparse. This study sought to determine the lifetime, 12-months period and point prevalence of work-related musculoskeletal disorders (WMSDs); the associated job risk factors and the coping strategies toward reducing the risk among nurses from selected hospitals in Ibadan, South-west Nigeria

**Methods:**

A previously validated self administered questionnaire which sought information on demographics, prevalence and pattern of WMSDs, associated job risk factors and coping strategies was employed as the survey instrument. A total of 160 questionnaires were distributed to nurses in the different hospitals but 128 questionnaires were returned yielding an 80% response rate. 10 of the returned questionnaires were excluded because of incomplete data.

**Results:**

Eighty-four point four percent of the nurses have had WMSDs once or more in their occupational lives. The 12-months period and point prevalence rate of WMSDs at any body region was 78% and 66.1% respectively. WMSDs occurred mostly in low back (44.1%), neck (28.0%), and knees (22.4%). 30.3% treated themselves or had visited other health practitioners for care. Nurses with > 20 years of clinical experience are about 4 times more likely to develop WMSDs (OR 3.81; CI 1.08-13.4) than those with 11-20 years experience. Working in the same positions for long periods (55.1%), lifting or transferring dependent patients (50.8%) and treating an excessive number of patients in one day (44.9%) were the most perceived job risk factors for WMSDs. Getting help in handling heavy patients (50.4%), modification of nursing procedures in order to avoid re-injury (45.4%), and modifying patient's/nurse position (40.3%) were the top three coping strategies.

**Conclusions:**

A high proportion of Nigerian nurses reported WMSDs at some body site in their occupational lives with the low back being injured most often. Education programmes on prevention and coping strategies for musculoskeletal disorders are recommended for nurses in order to reduce the rate of occupational hazards and also promote efficiency in patient care.

## Background

The term musculoskeletal disorders encompass a gamut of inflammatory and degenerative conditions that affects the muscles, tendons, ligaments, joints, peripheral nerves, and supporting blood vessels with consequent ache, pain or discomfort [1,2]. Musculoskeletal disorders are reported to occur in certain industries and occupations with rates up to three or four times higher than the average rate across all industries [1]. Work-related musculoskeletal disorders (WMSDs) are defined as musculoskeletal disorders that results from a work-related event [3]. WMSDs are common among health care workers, with the nursing population that constitutes about 33% of the hospital workforce at particularly high risk and accounting for 60% of the reported occupational injuries [4-6]. WMSDs are reported to significantly impact on quality of life [1], cause lost work time or absenteeism, increase work restriction, transfer to another job [7-9], or disability than any other group of diseases [10-12] with a considerable economic toll on the individual, the organization and the society as a whole [13].

A number of intrinsic and extrinsic factors have been implicated in the aetiology of WMSDs [1,14-18]. Silverstein et al [19] reported repetitious movement, awkward postures, and high force levels as the three primary risk factors that have been associated with WMSDs. Nurses routinely perform activities that require lifting heavy loads, lifting patients, working in awkward postures, and transferring patients out of bed and from the floor. These work tasks put nurses at high risk for acute and cumulative WMSDs. Nursing has suffered severe brain drain like most other health professions in Nigeria in the recent years. Many Nigerian nurses have emigrated to Europe, northern hemispheric countries and the oil rich middle-east in search of better life and condition of service. This has contributed to the problem of inadequate staffing, and this has been associated with WMSDs among nurses [20-22]. Because nurses are already at risk for musculoskeletal disorders, a reduction in professional nursing staff and other changes in nursing care delivery are likely to lead to even higher rates of these disorders [23]. Although several authors have reported the prevalence of WMSDs among nurses in the developed populations [5-7,15,24], however, data on prevalence of WMSDs are limited in Sub-Sahara Africa for referencing. This study sought to determine the lifetime, 12-months period and point prevalence of WMSDs; the associated job risk factors and the coping strategies toward reducing the risk for development of WMSDs among nurses from selected hospitals in Ibadan, South-west Nigeria.

## Methods

A survey was conducted on prevalence, work factors and coping strategies of WMSDs among nurses in Nigeria. Respondents for the study were nurses in active service from three selected hospitals in Ibadan. Namely, (1) University College Hospital which is the apex tertiary hospital in Nigeria, representing the federal government owned hospitals, (2) Adeoyo General Hospital, representing the state government owned hospitals and (3) Oluyoro Catholic Hospital representing the private owned hospitals. University of Ibadan/University College Hospital joint Institutional Review Committee gave approval for this study and all the respondents gave their signed consent.

A four section questionnaire was employed as the survey instrument. Section A sought information on demographic profile such as such as age, height, weight, and gender. Section B was on occupational health in nursing practice and sought to elicit general information on years of practice, work status, work setting, practice specialty, patient population, and nursing activities. The symptom-survey segment of the occupational health in nursing practice section was a modification of the standardized Nordic questionnaire [25] and consisted of questions referring to nine body areas. These are 3 upper limb segments (Shoulders, elbows, wrists/hands/thumb), 3 lower limb segments (Hips/thighs, knees, ankles/feet), and 3 trunk segments (Neck, upper back and lower back). Its section C contained items on perceptions on job risk factors that may contribute to development of work-related musculoskeletal disorders while section D gleaned data on coping strategies toward reducing the risk for development of work-related musculoskeletal disorders among nurses. The questionnaire for this study was adapted from previously validated questionnaire on WMSDs among physical therapist [26,27] (See additional file 1).

A total of 160 questionnaires were distributed to the different hospitals but 128 questionnaires were returned yielding an 80% response rate. However, 92.2% (118) of the returned questionnaires was used in the data analyses and the others (10) were excluded because of incomplete data.

### Data analyses

Data were summarized using the descriptive statistics of mean, standard deviation and percentages. Pearson's Chi-square analysis was used to determine the association of prevalence of self-reported musculoskeletal symptoms with personal characteristics and job risk factors. Using 2 × 2 contingency tables, odds ratios (OR) and upper and lower 95% confidence intervals (CI) were calculated to estimate the relative risk of WMSDs. The data analyses were carried out using SPSS 13.0 version software (SPSS Inc., Chicago, Illinois, USA). The α level was set at 0.05.

## Results

The mean age, height weight and body mass index of the respondents were 36.4 ± 7.75 years, 1.60 ± 0.07 m, 66.8 ± 11.2 Kg and 26.2 ± 4.63 Kg/m^2 ^respectively. The descriptive information of all respondents is presented in Table 1. Women and men accounted for 97.5% and 2.5% of the sample population respectively. Hospitals were found to be the most common work setting among the respondents as 90.7% of the population works in the clinical setting (81.3% in the federal hospital, 15.3% in the state hospital and 3.4% in the private owned hospital), 2.5% in the academia and 6.8% in the public health sector. 57.6% of the respondents were staff nurses, 11.6% were senior nursing officers, 10.8% were principal nursing officers, 10.2% were chief nursing officers, 4.9% were chief public health officers, 2.4% were matrons while 2.5% were assistant directors of nursing services.

**Table 1:** Descriptive information of all respondents to questionnaire

	Range		
Variable	minimum	maximum	Mean ± S.D
Age (yrs)	22.0	58.0	36.4 ± 7.752
Height (m)	1.40	1.90	1.60 ± 0.07
Weight (Kg)	45.0	100.0	66.8 ± 11.2
Body mass index (Kg/m^2^)	15.6	43.4	26.2 ± 4.63
Years of experience (yrs)	1.00	33.0	11.8 ± 7.56
Hours per week (hrs)	10.0	58.0	40.4 ± 6.51

Of the respondents, 84.4% reported that they had experienced work-related musculoskeletal pain or discomfort at some time in their occupational lives. The respondents reported a 12-month prevalence rate of WMSDs at any body region to be 78%. The point prevalence rate of WMSDs was 66.1% for all the respondents. Table 2 show**s **that the 12-months prevalence rates of WMSDs was highest in the low back (44.1%), followed by the neck (28.0%) and then knees (22.4%) but least in the hips/thighs (3.4%). Of all the respondents that indicated WMSDs only 30.3% reported that they had treated themselves or had sought treatment from other health practitioners for WMSDs. Of the respondents who reported WMSDs, a variable number reported having visited a health practitioner for treatment, with 40% of those with shoulder, 60.0% of those with upper back, 40.4% of those with low back, 50.0% of those with wrists/hands, 25.0% of those with knees, and 25.0% of those with ankles/feet problems respectively.

**Table 2:** Prevalence of work-related musculoskeletal disorders in the different body regions

Body region	frequency	percentage (%)
Low back	52	44.1
Neck	33	28.0
Knees	26	22.4
Upper back	20	16.8
Wrists/Hands	19	16.2
Shoulder	15	12.6
Ankles/Feet	12	10.2
Elbow	8	7.1
Hips/Thighs	4	3.4

Table 3 shows the association between self-reported 12-months prevalence of WMSDs and age among all the respondents. The prevalence rate of WMSDs increased with increasing age but was lowest in the respondents who were over 50 years of age. Highest percentage of the respondents (24.4%) experienced their first episode of WMSDs in the first five year of clinical practice, 20.2% as student nurses, and 19.3% during 5-15 years of nursing practice while 1.7% had their first episode of WMSDs before training as a nurse. Most of the respondents (54.6%) reported WMSDs of gradual onset, 20.2% reported WMSDs of sudden onset while only 2.5% implicated a known accident. WMSDs led to change of specialty from the clinical setting to public health in 5.0% of the respondents, while 0.8% reported they considered leaving the nursing profession to pursue another career as a result of WMSDs. Only 33.6% of the respondents had some form of training on ergonomics.

**Table 3:** Association of self-reported 12-month prevalence of work-related musculoskeletal disorders and age among all the respondents

Age category (yrs)	frequency	percentage	χ^2^	p-value
21-30	19	59.4	3.732	0.292
31-40	39	68.4		
41-50	15	71.4		
> 50	3	37.5		

Level of significance was set at p < 0.05.

The number of hours per week a nurse spent in direct patient care is averaged 40.4 ± 6.51 hours. However, the number of hours per week was not significantly different (F = 0.009; p = 0.991) across the clinical work settings. The rates of WMSDs was not significantly associated with age (χ^2 ^= 33.919; p = 0.329), gender (χ^2 ^= 1.297; p = 0.255), number of years in nursing practice (χ^2 ^= 36.83; p = 0.295), or number of hours per week in direct patient care (χ^2 ^= 22.905; p = 0.262). Table 4 shows the association of the difference in clinical years of practice and the risk for WMSDs among all the respondents with the odds ratio [OR] and 95% confidence interval [CI]. The OR and 95% [CI] for WMSDs among nurses with > 20 years of clinical experience compared those with 11-20 years and those with 1-10 years of clinical years of clinical experience were (OR 3.81; CI 1.08-13.4) and (OR 1.78; CI 0.58-5.53) respectively. However, the association between clinical years of experience and the prevalence of WMSDs among all the respondents was not significant (p > 0.05).

**Table 4:** Association of the difference between categories of years of clinical practice and the risk of WMSDs among all the respondents

				[Odds Ratio (95% Confidence interval)]		
Group	No. (%)	χ^2^	p-value	A - B	A - C	B-C
A	39 (60.9%)	5.060	0.080	[0.47(0.19-1.15)]	[1.78(0.58-5.53)]	[(3.81(1.08-13.4)]
B	30(76.9%)					
C	7 (46.7%)					

Level of significance was set at p < 0.05.

Key:

Group A: 1-10 years of clinical practice

Group B: 11-20 years of clinical practice

Group C: >20 years of clinical practice

Table 5 show**s **the respondents' perceptions of the risk factors that could contribute to work-related discomfort and injury. The respondents who had experienced WMSDs indicated that working in the same positions for long periods (55.1%), lifting or transferring dependent patients (50.8%) and treating an excessive number of patients in one day (44.9%) were the most perceived job risk factors precipitating WMSDs during their clinical practice. Table 6 showed the percentage indicating the coping strategies of the respondents toward reducing the risk for development of WMSDs. For each item of the coping strategies section of the questionnaire, the percentages that indicate the most appropriate response were summarized. Getting help in handling heavy patients (50.4%), modification of nursing procedures in order to avoid stressing an injury (45.4%), and modifying patient's/nurse position (40.3%) were the top three coping strategies indicated by the respondents in ameliorating the risk of WMSDs.

**Table 5:** Percentage indicating respondents' perceptions of job risk factors that may contribute to development of work-related musculoskeletal disorders among all respondents (Greater than 7 on a scale of 0 - 10)

Job risk factor	Percentage
1. Working in the same positions for long periods (Standing, bend over, sitting, kneeling)	55.1
2. Lifting or transferring dependent patients	50.8
3. Bending or twisting your back in an awkward way	45.8
4. Treating an excessive number of patients in one day	44.9
5. Carrying, lifting, or moving heavy materials or equipment (e.g., continuous passive motion machines)	42.4
6. Performing manual orthopaedic techniques (Joint mobilizations, soft tissue mobilization)	40.0
7. Not enough rest breaks or pauses during the workday	39.0
8. Work scheduling (Overtime, irregular shifts, length of workday)	33.9
9. Working in awkward and cramped positions	33.1
10. Continuing to work while injured or hurt	32.2
11. Reaching or working away from your body	31.6
12. Unanticipated sudden movement or fall by patient	28.8
13. Inadequate training on injury prevention	27.1
14. Working near or at your physical limits	23.7
15. Working with confused or agitated patients	16.0
16. Performing the same task over and over	14.4
17. Assisting patients during gait activities	12.7

**Table 6:** Percentage indicating the coping strategies of the respondents toward reducing the risk for development of work-related musculoskeletal disorders

Strategies	Percentage
1. I get someone to help me handle a heavy patient	50.4
2. I modify my nursing procedure in order to avoid stressing an injury	45.4
3. I modify patient's position/my position	40.3
4. I stop a treatment if it causes or aggravate my discomfort	33.6
5. I warm up and stretch before performing my nursing duties	32.8
6. I select techniques/procedures that will not aggravate or provoke my discomfort	30.5
7. I adjust plinth/bed height so I can stretch and change posture	21.8
8. I use different part of my body for ease in administering my nursing procedure	20.2
9. I pause regularly so I can stretch and change posture	14.3

## Discussion

Studies have shown that musculoskeletal problems are particularly common in health care workers who are in direct contact with patients [7,23]. Reports from other populations have shown that nurses, nursing aides, and orderlies have the highest rates of WMSDS in the medical industry [28,29]. The high prevalence of musculoskeletal disorders among nurses is thought to be due to physical work demands, as well as to work organizational factors, of which scheduling is an important component [28,30,31]. However, there is a dearth of studies on WMSDs among nurses in Nigeria.

The results of this study indicated a lifetime prevalence of 84.4% among the nurses. The 12-months and point prevalence of WMSDs at any body region were 78% and 66.1% respectively. The prevalence rates of WMSDs in nurses have varied according to studies but have been generally high. In a previous study from Nigeria, Fabunmi et al [32] reported the 12 months period prevalence of self reported musculoskeletal disorders at any body site to be 90.7%. Smith et al [33] in a study from rural Japan reported a 12-month prevalence of 91.9%. In a study conducted in the US, Josephson [34] reported a prevalence of 72.5% while Harber et al [24] reported that 52% of nurses reported experiencing work-related back pain within a 6-month period. Prevalence of musculoskeletal disorders has been noted to vary across occupational groups and over national boundaries [33]. Subjectivity of terms, variations in instrument, organizational differences in work settings, and cultural differences in the perception and reporting of pain and disorders are adduced for the variation in rates of WMSDs in the different studies.

The highest prevalence of 12 months period WMSDs in nurses according to body sites in this study was the low back (44.1%), followed by the neck (28.0%) and then knees (22.4%). This distribution pattern is consistent with literature. LBP is the most common musculoskeletal disorder in adult and about 60-80% of all individuals will experience the condition at some stage in their lifetime [35]. LBP is one of the most important WMSDs among nursing professionals, accounting for a point prevalence of approximately 17%, an annual prevalence of 40-50% and a lifetime prevalence of 35-80% [36]. Some researchers reported that more than half (56%) of their nurses have ongoing back troubles [37]. However, previous studies have documented various rates of work-related low back pain (LBP) in nurses from various populations for a 12-month time period: Smith et al [38] Korea 19.8%, Yip [17] Hong Kong 40.6%, Limpscomb et al [23] USA 29.0%, Niedhammer et al [39] France 41.1%, Smedley et al [15] England 45.0%, Smith and Leggat [29] Australia 59.0%, Josephson et al [34] Sweden 64.0%, and Fabunmi et al [32] Nigeria 79.4%.

Lifting patients in bed, transferring patients out of bed, and lifting patients from the floor were the job activities most commonly reported as sources of back pain among nurses [15,24]. Studies in biomechanics have also implicated factors such as physical loading, body flexion, rotation and weight loading in the aetiology of prevalent occupational LBP. Our finding on the high prevalence of work-related neck and knee pain among nurses is consistent with the pattern reported in literature. The neck and the knees have been recognized as common body sites of WMSDs among health care practitioners [29,33,40,41].

A high percentage of the nurses in this study experienced their first episode of WMSDs in the first five year of clinical practice. Our results suggest that WMSDs increase with age and duration of employment respectively. It was observed that after age 50 years and at greater than 20 years of clinical practice, the prevalence of WMSDs declined. The lower rate of WMSDs among the very senior nurses in terms of age and years of clinical practice may be attributed to less patient handling but more administrative duties that often come with rise in job cadre. Another explanation might be that experienced and older nurses have increased level of knowledge about injury prevention, avoid harmful physical load, and have developed better coping strategies for musculoskeletal problems than the less experienced and younger nurses. Survivor effect was also implicated for the inverse trend observed between lower prevalence of WMSDs and each of older age and higher clinical experience. From occupational studies, healthy survivor effect describes a continuing selection process such that those who remain in an employment tend to be healthier over time. The healthy worker survivor effect generally attenuates an adverse effect of exposure [42]. It is believed in cross-sectional studies, that survivor effects will typically decrease the observed associations between symptomatic disorders and physically demanding jobs [43]. However, the mechanism of the survivor effect is still poorly understood.

From this study, the OR and 95%CI results indicate that the relative risk of WMSDs is about 4 times more among nurses with greater than 20 years of clinical experience than those with 11-20 years and are about 2 times more in those with 1-10 years of clinical years of experience respectively. However, this result may be due to chance rather than true effect as Chi square test of association result did not reveal significant association between rates of WMSDs and each of age and number of years of clinical practice. From the study population, 30.3% of the nurses in this study have sought treatment for their musculoskeletal disorders. Nurses with upper and lower back WMSDs sought treatment more than those with WMSDs in other body sites.

Working in the same positions for long periods, lifting or transferring dependent patients and treating an excessive number of patients in one day were the most perceived job risk factors precipitating WMSDs among the nurses in this study. These findings are consistent with previous reports indicating manual patient handling, transferring or moving as important predictors of musculoskeletal disorders and low back pain among nurses [14-17,29]. Wilkinson et al [6] and Harber et al [24] implicated lifting patients as the most common mechanism for musculoskeletal disorders among nurses. Alexopoulos et al [18] reported that handling of physical loads among nurses seems to put them at risk for the occurrence of musculoskeletal disorders. Ando et al [44] also suggested that musculoskeletal pain among hospital nurses may have associations with some actual tasks and items related to work postures, work control, and work organization.

From this study, getting assistance or support staff in handling heavy patients, modification of nursing procedures in order to avoid re-injury or stressing an injury, and modification of patient's/nurse position were the top three coping strategies in ameliorating the risk of WMSDs. These coping strategies among Nigerian nurses seem similar to previous findings. Workers performing strenuous work are often advised to prevent problems and to cope with musculoskeletal symptoms by changing their working technique, using lifting equipment, taking breaks, and avoiding strenuous work tasks [45-47]. This is also similar to the submission of Lambert and Lambert [48] on methods for fostering effective coping strategies. Less than half of those with WMSDs visited other health practitioners for treatment or engaged in self-treatment. It can be adduced that those who sought medical care represent the more severe cases and the more serious pathology. However, this study did not assess the severity of pain or discomfort from WMSDs of the respondents.

### Limitations of the study

This study is limited in its generalizability because of the non-probability sample employed. However, we tried to minimize this effect, by systematically selecting three of the Nigeria's leading and biggest hospitals in Ibadan, Nigeria each one representing the different tiers of health care providers. The variability of the workload and ergonomics knowledge of the study respondents in these different hospitals may influence homogeneity. This study investigated the lifetime and 12 months period experience of WMSDs which could also lead to some degree of misclassification due to recall bias. Like all other cross-sectional or self-report studies, it is possible that our respondents might have given vague answers or exaggerated their WMSDs. It is also possible that some of the respondents in our study perceived their musculoskeletal disorders as WMSDs regardless of whether they were caused by work or not. Adegoke et al [27] posited that work may only be a contributory factor in the aetiology of musculoskeletal disorders among workers and that it may be difficult to distinguish between WMSDs and musculoskeletal disorders since their consequences in response to work demands may be similar. This study was delimited to nurses in active service only, those who left the workforce due to retirement or WMSDs or any other reason were not included in the current analysis. A prospective cohort study design with larger sample size is warranted in the future to provide more sound research evidence on WMSDs and healthy survivor effects among nurses in Nigeria.

## Conclusion

A high proportion of Nigerian nurses reported WMSDs at some body site in their occupational lives with the low back being injured most often. The knowledge of ergonomics was generally poor among the nurses. Working in the same positions for long periods, lifting or transferring dependent patients and treating an excessive number of patients in one day were the most perceived job risk factors for WMSDs. While getting help in handling heavy patients, modification of nursing procedures in order to avoid stressing an injury, and modifying patient's/nurse position were the top three coping strategies. Less than half of those with WMSDs visited other health practitioners for treatment or engaged in self-treatment. We recommend that education programmes on prevention and coping strategies for musculoskeletal disorders be made mandatory for nurses in order to reduce the rate of WMSDs among them and to promote efficiency in patient care.

## Competing interests

The authors declare that they have no competing interests.

## Authors' contributions

BMS conceived the idea for this study, participated in the design of methodology and data collection and drafted the manuscript. CE participated in the design of the study's methodology, conducted the analysis and interpretation of data and prepared the final manuscript for publication. AL developed the study's methodology, participated in data acquisition and drafted the manuscript. AA participated in the design of the study's methodology and data acquisition and drafted the manuscript.

All authors read and approved the final manuscript.

## Pre-publication history

The pre-publication history for this paper can be accessed here:

http://www.biomedcentral.com/1471-2474/11/12/prepub

## Supplementary Material

Additional file 1 **Survey on work-related musculoskeletal disorders among Nigerian nurses**. The survey questionnaire sought information on demographics, prevalence and pattern of work-related musculoskeletal disorders, associated job risk factors and coping strategies among Nigerian nurses. Click here for file
