# Supplementary material for: Work-Related Musculoskeletal Disorders among Nurses in Ibadan, South-west Nigeria: a cross-sectional survey
Source: BMC Musculoskelet Disord. 2010 Jan 20;11:12. doi: 10.1186/1471-2474-11-12 (PMC2823665; doi:10.1186/1471-2474-11-12)
Supplement: Additional file 1 — Survey on work-related musculoskeletal disorders among Nigerian nurses. The survey questionnaire sought information on demographics, prevalence and pattern of work-related musculoskeletal disorders, associated job risk factors and coping strategies among Nigerian nurses. [file 1471-2474-11-12-S1.DOC]

**SURVEY ON WORK-RELATED MUSCULOSKELETAL DISORDERS AMONG NIGERIAN NURSES**

You are hereby invited to participate in a study designed to obtain information on work-related musculoskeletal disorders among Nigerian nurses. The survey hopes to investigate job-related factors leading to musculoskeletal disorders in nursing practice, its prevalence and your coping strategies. The findings from this study may help to understand prevention and coping strategies for musculoskeletal disorders among nurses in order to reduce the rate of occupation hazards and also improve patients’ care.

All information obtained is for research purpose only and will be treated with utmost confidentiality. Your participation in this study is voluntary and your signed consent is required, you also have the right to withdraw from the study at any time you choose to. If you have any question regarding this survey, please feel free to ask or contact us at the department of physiotherapy, College of Medicine, University of Ibadan, Ibadan, Nigeria.

We will greatly appreciate your help in responding to participate in this study.

Thank you for your cooperation and assistance.

**Consent:** Now that the study has been explained to me and I understand the purpose. I will be willing to take part in the study.

…………………………………………….. … ………………………..

Signature of Participant/Date Signature of researcher/Date

**SECTION A. Questions on demographic data**

1. What is your age (as at last birthday)? …………………………….
2. What is your height (m)?...................................................................
3. What is your weight (Kg)?................................................................
4. What is your sex?..............................................................................
5. What best describe your current area of practice?
   1. Clinic…………………………….
   2. Academic………………………….
   3. Public health………………………
   4. Others……………………………..
6. What is your rank/cadre……………………………………………..
7. What is the year of graduation from nursing school?..........................
8. How many years of professional experience do you have?.................
9. Do you have a post basic nursing qualification? Yes……… No………..
10. What is your work status in the last 12 months? Full time………Part time……….
11. Please indicate your work setting
    1. Tertiary…………
    2. Secondary………
    3. Primary…………
    4. Others…………..
12. Please indicate the number of hours per week (HPW) you spend in direct patient care in the last 12 months as part of your nursing practice…………………………

**SECTION B. Occupational health in nursing practice**

13. Have you ever experienced work-related ache, pain, discomfort, or injury that lasted for more than three days (not within the previous 12 months) in your nursing career till date? Yes …………. No……………..

14. Have you ever experienced work-related ache, pain, discomfort, or injury in any part(s) of your body that lasted for more than three days in the last 12 months?

Yes…………………… No……………………..

15. If you answered ‘Yes’ to Question 14, please check all that apply

i. Neck……………………………………………

ii. Shoulder………………………………………

iii. Upper back (thoracic)…………………………

iv. Elbow/forearm………………………………..

v. Low back (lumbar/sacral)………………………

vi. Wrist/hand…………………………………….

vii. Thumb………….…………………………….

vii. Hips/Thighs…………………..………………

viii. Knees…………………………………………

ix. Ankles…………………………………………

16. If you answered ‘Yes’ to Question 14, please indicate one most significant body location/site as stated in Question 15 where you have experienced work-related problems………………………………………………………..

17. When did you first experience this work-related problem?

i. Before training as a nurse………………………..

ii. As a student nurse………………………………

iii. In the first five years after graduation………….

iv. 5-15 years after graduation……………………..

v. >15 years after graduation………………………

vi. Don’t know……………………………………..

18. What was the onset of work-related problem like?

1. Gradual…………………………………………..
2. Sudden……………………………………………
3. As a result of an accident………………………...

19. Have you ever treated yourself or sought treatment from any health professional

as a result of work-related problem? Yes…………….. No……………………

20. Have you ever changed the area/specialty of nursing practice as a result of work-

related problem? Yes……………………….. No………………………………

21. If you answered ‘Yes’ to Question 19, what did you change from?

i. From…………………………..

ii. To…………………………….

22. Have you ever had training on ergonomics or how to prevent occupational

hazards? Yes………………………….. No……………………………..

23. Indicate how you may possibly reduce strain on your body when carrying out

your nursing duties. Please tick all that apply.

1. I will prefer to use adjustable bed/plinth……………………
2. I will prefer to use sliding board……………………………
3. I will prefer to use lifting belt………………………………
4. I will prefer to use splints…………………………………..
5. Others (indicate)……………………………………………
6. None of the above………………………………………….

24. Have ever left the nursing profession to pursue another career as a result of work-

related disorders?

1. Yes…………………………
2. No………………………….

**SECTION C: Perceptions on job risk factors that may contribute to development of work-related musculoskeletal disorders**

Listed below are 17 conditions and tasks at work that could contribute to job-related problems. Please indicate, on a scale of 0 to 10, how much of a problem (if any) each item is for you by circling the appropriate number." A score of 0 to 1 was equivalent to a job factor being "no problem," a score of 2 to 7 was rated as a "minimal to moderate problem," and a score of 8 to 10 indicated that a job factor was considered a "major problem.

**Job risk factor**

_______________________________________________________________________

1. Performing the same task over and over

|  |  |  |  |  |  |  |  |  |  |  |
| --- | --- | --- | --- | --- | --- | --- | --- | --- | --- | --- |
| 0 1 2 3 4 5 6 7 8 9 10 | | | | | | | | | | |

2. Treating an excessive number of patients in one day

|  |  |  |  |  |  |  |  |  |  |  |
| --- | --- | --- | --- | --- | --- | --- | --- | --- | --- | --- |
| 0 1 2 3 4 5 6 7 8 9 10 | | | | | | | | | | |

3. Performing manual orthopaedic techniques

(Joint mobilizations, soft tissue mobilization)

|  |  |  |  |  |  |  |  |  |  |  |
| --- | --- | --- | --- | --- | --- | --- | --- | --- | --- | --- |
| 0 1 2 3 4 5 6 7 8 9 10 | | | | | | | | | | |

4. Not enough rest breaks or pauses during the workday

|  |  |  |  |  |  |  |  |  |  |  |
| --- | --- | --- | --- | --- | --- | --- | --- | --- | --- | --- |
| 0 1 2 3 4 5 6 7 8 9 10 | | | | | | | | | | |

5. Working in awkward and cramped positions

|  |  |  |  |  |  |  |  |  |  |  |
| --- | --- | --- | --- | --- | --- | --- | --- | --- | --- | --- |
| 0 1 2 3 4 5 6 7 8 9 10 | | | | | | | | | | |

6. Working in the same positions for long periods

(Standing, bend over, sitting, kneeling)

|  |  |  |  |  |  |  |  |  |  |  |
| --- | --- | --- | --- | --- | --- | --- | --- | --- | --- | --- |
| 0 1 2 3 4 5 6 7 8 9 10 | | | | | | | | | | |

7. Bending or twisting your back in an awkward way

|  |  |  |  |  |  |  |  |  |  |  |
| --- | --- | --- | --- | --- | --- | --- | --- | --- | --- | --- |
| 0 1 2 3 4 5 6 7 8 9 10 | | | | | | | | | | |

8. Working near or at your physical limits

|  |  |  |  |  |  |  |  |  |  |  |
| --- | --- | --- | --- | --- | --- | --- | --- | --- | --- | --- |
| 0 1 2 3 4 5 6 7 8 9 10 | | | | | | | | | | |

9. Reaching or working away from your body

|  |  |  |  |  |  |  |  |  |  |  |
| --- | --- | --- | --- | --- | --- | --- | --- | --- | --- | --- |
| 0 1 2 3 4 5 6 7 8 9 10 | | | | | | | | | | |

10. Continuing to work while injured or hurt

|  |  |  |  |  |  |  |  |  |  |  |
| --- | --- | --- | --- | --- | --- | --- | --- | --- | --- | --- |
| 0 1 2 3 4 5 6 7 8 9 10 | | | | | | | | | | |

11. Lifting or transferring dependent patients

|  |  |  |  |  |  |  |  |  |  |  |
| --- | --- | --- | --- | --- | --- | --- | --- | --- | --- | --- |
| 0 1 2 3 4 5 6 7 8 9 10 | | | | | | | | | | |

12. Working with confused or agitated patients

|  |  |  |  |  |  |  |  |  |  |  |
| --- | --- | --- | --- | --- | --- | --- | --- | --- | --- | --- |
| 0 1 2 3 4 5 6 7 8 9 10 | | | | | | | | | | |

13. Carrying, lifting, or moving heavy materials or

equipment (e.g., continuous passive motion machines)

|  |  |  |  |  |  |  |  |  |  |  |
| --- | --- | --- | --- | --- | --- | --- | --- | --- | --- | --- |
| 0 1 2 3 4 5 6 7 8 9 10 | | | | | | | | | | |

14. Unanticipated sudden movement or fall by patient

|  |  |  |  |  |  |  |  |  |  |  |
| --- | --- | --- | --- | --- | --- | --- | --- | --- | --- | --- |
| 0 1 2 3 4 5 6 7 8 9 10 | | | | | | | | | | |

15. Assisting patients during gait activities

|  |  |  |  |  |  |  |  |  |  |  |
| --- | --- | --- | --- | --- | --- | --- | --- | --- | --- | --- |
| 0 1 2 3 4 5 6 7 8 9 10 | | | | | | | | | | |

16. Work scheduling (Overtime, irregular shifts,

length of workday)

|  |  |  |  |  |  |  |  |  |  |  |
| --- | --- | --- | --- | --- | --- | --- | --- | --- | --- | --- |
| 0 1 2 3 4 5 6 7 8 9 10 | | | | | | | | | | |

17. Inadequate training on injury prevention

|  |  |  |  |  |  |  |  |  |  |  |
| --- | --- | --- | --- | --- | --- | --- | --- | --- | --- | --- |
| 0 1 2 3 4 5 6 7 8 9 10 | | | | | | | | | | |

**SECTION D: Coping strategies toward reducing the risk for development of work-related musculoskeletal disorders**

The response to the following statements should reflect what you actually do in practice rather than what you would like to do or think you should do.

In order to reduce the strain on my body when working

|  | Strategies | Almost Always | Sometimes | Almost Never |
| --- | --- | --- | --- | --- |
| 1 | I get someone else to help me handle a heavy patient |  |  |  |
| 2 | I modify patient’s position/ my position |  |  |  |
| 3 | I use a different part of my body in administering my nursing procedure |  |  |  |
| 4 | I warm up and stretch before performing my nursing duties |  |  |  |
| 5 | I modify my nursing procedure in order to avoid stressing an injury |  |  |  |
| 6 | I pause regularly so I can stretch and change posture |  |  |  |
| 7 | I adjust plinth/bed height so I can stretch and change posture |  |  |  |
| 8 | I select techniques/procedures that will not aggravate or provoke my discomfort |  |  |  |
| 9 | I stop a treatment if it causes or aggravate my discomfort |  |  |  |

Thank you for your assistance
